# Supplementary figures and images for: Beyond ranking nodes: Predicting epidemic outbreak sizes by network centralities
Source: PLoS Comput Biol. 2020 Jul 22;16(7):e1008052. doi: 10.1371/journal.pcbi.1008052 (PMC7398553; doi:10.1371/journal.pcbi.1008052)

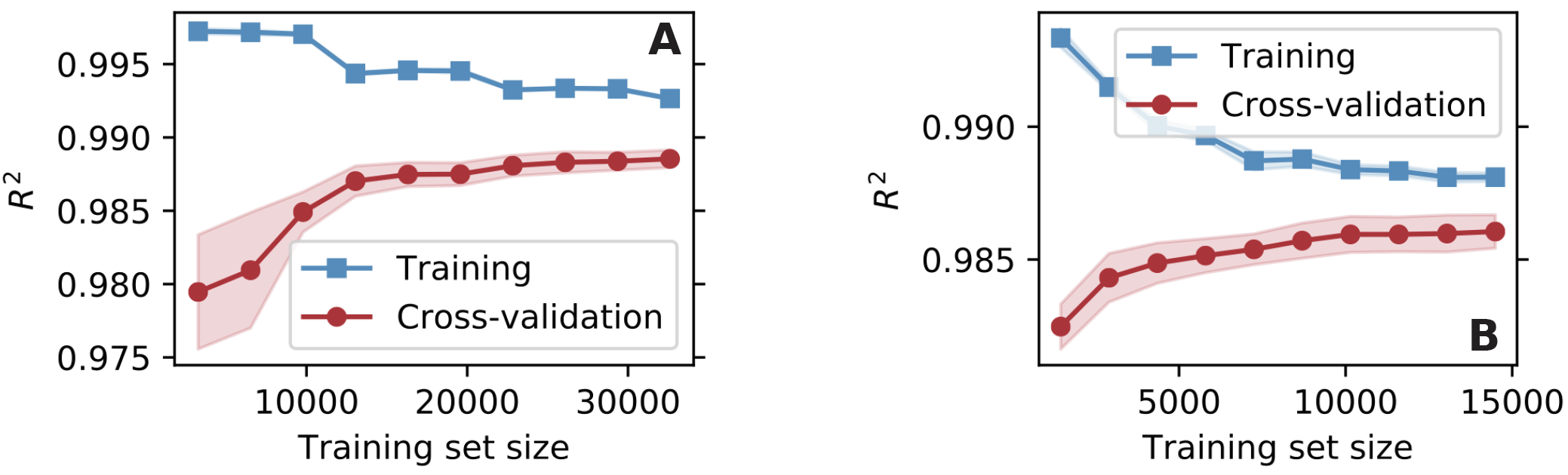

Supplement: S1 Fig — Panel A shows the curves for the most predictive pair of centralities (degree and PageRank); panel B shows the curves for density and PageRank. (TIF) [file pcbi.1008052.s001.tif]

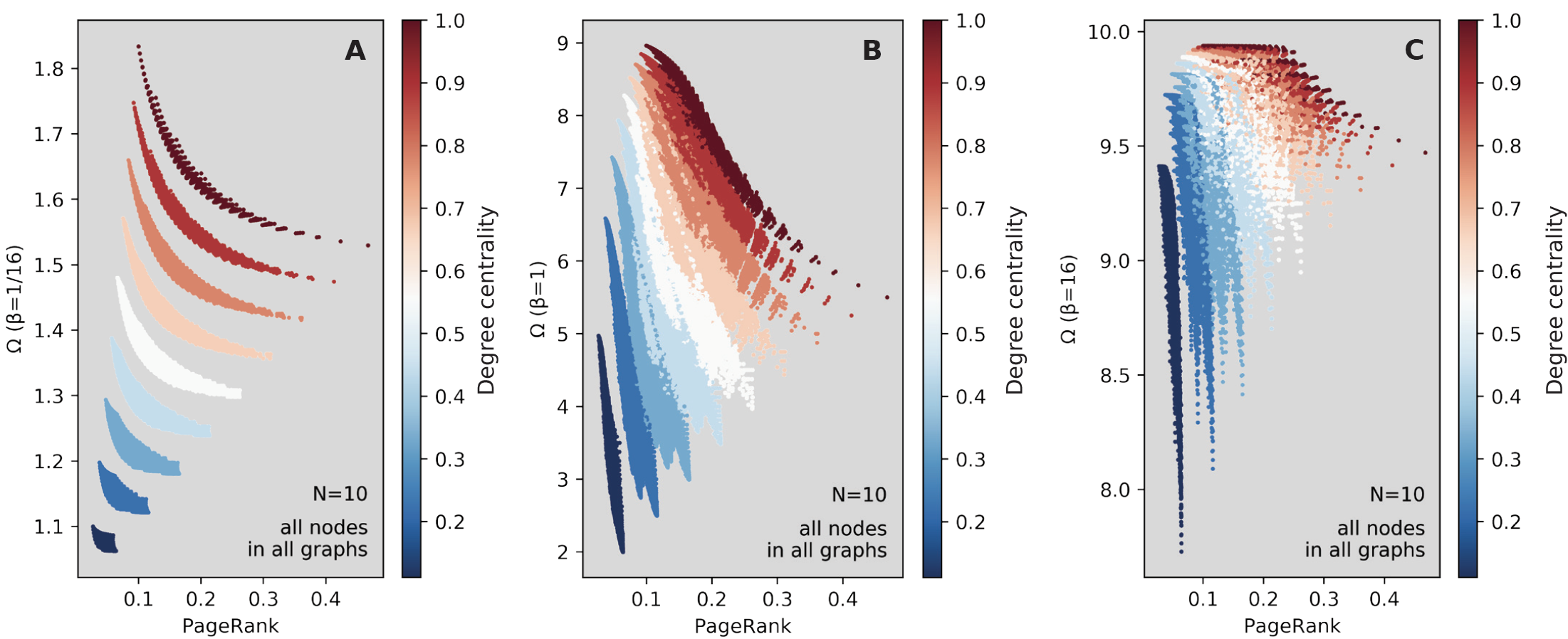

Supplement: S2 Fig — Corresponding to Fig 4 but for PageRank instead of Eigenvector centrality. (TIF) [file pcbi.1008052.s002.tif]

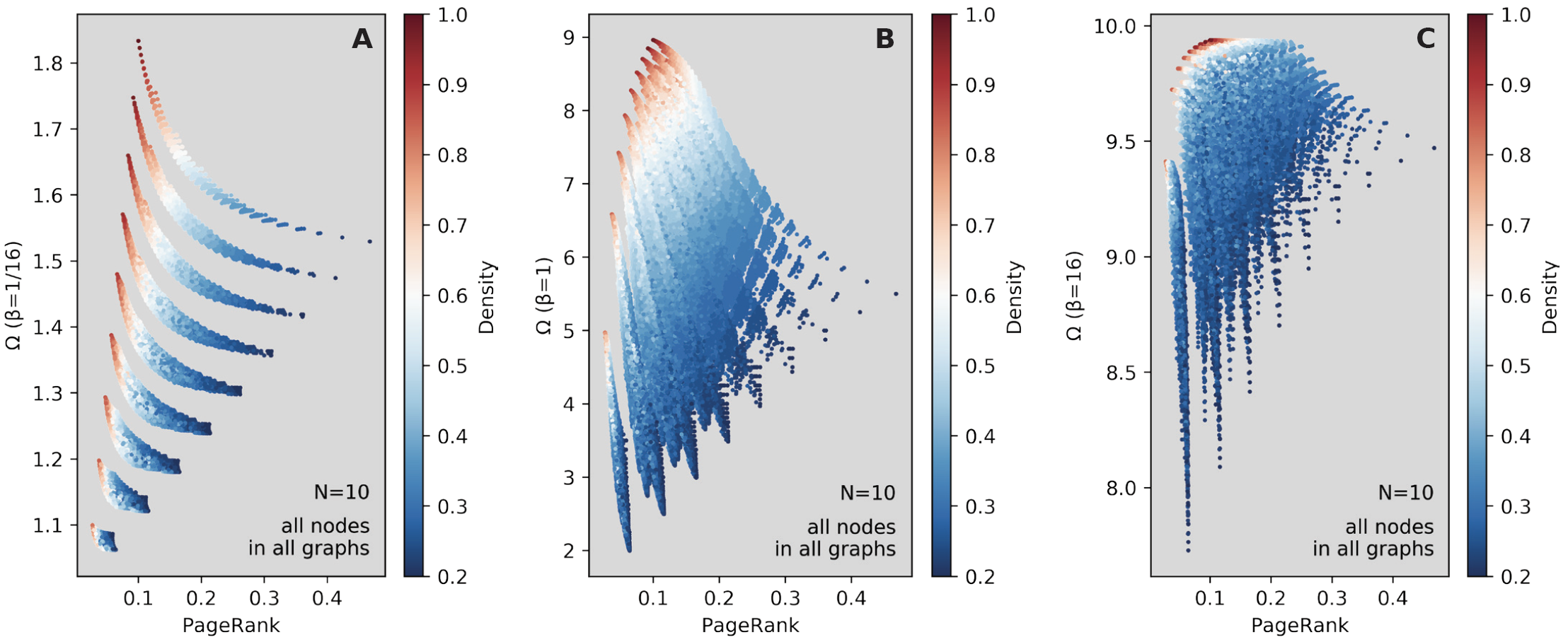

Supplement: S3 Fig — Corresponding to Fig 5 but for PageRank instead of Eigenvector centrality. (TIF) [file pcbi.1008052.s003.tif]
